# A grass–fire cycle eliminates an obligate-seeding tree in a tropical savanna

**DOI:** 10.1002/ece3.1285

**Published:** 2014-10-14

**Authors:** David M J S Bowman, Harry J MacDermott, Scott C Nichols, Brett P Murphy

**Affiliations:** 1 School of Biological Sciences, University of TasmaniaPrivate Bag 55, Hobart, TAS, 7001, Australia; 2 NERP Environmental Decisions Hub, School of Botany, The University of MelbourneParkville, VIC, 3010, Australia

**Keywords:** Alternate stable states, fire regime, grass–fire cycle, invasion ecology, obligate-seeder, tropical savanna

## Abstract

A grass–fire cycle in Australian tropical savannas has been postulated as driving the regional decline of the obligate-seeding conifer *Callitris intratropica* and other fire-sensitive components of the regional flora and fauna, due to proliferation of flammable native grasses. We tested the hypothesis that a high-biomass invasive savanna grass drives a positive feedback process where intense fires destroy fire-sensitive trees, and the reduction in canopy cover facilitates further invasion by grass. We undertook an observational and experimental study using, as a model system, a plantation of *C. intratropica* that has been invaded by an African grass, gamba (*Andropogon gayanus*) in the Northern Territory, Australia. We found that high grass biomass was associated with reduced canopy cover and restriction of foliage to the upper canopy of surviving stems, and mortality of adult trees was very high (>50%) even in areas with low fuel loads (1 t·ha^−1^). Experimental fires, with fuel loads >10 t·ha^−1^, typical of the grass-invasion front, caused significant mortality due to complete crown scorch. Lower fuel loads cause reduced canopy cover through defoliation of the lower canopy. These results help explain how increases in grass biomass are coupled with the decline of *C. intratropica* throughout northern Australia by causing a switch from litter and sparse perennial grass fuels, and hence low-intensity surface fires, to heavy annual grass fuel loads that sustain fires that burn into the midstorey. This study demonstrates that changes in fuel type can alter fire regimes with substantial knock-on effects on the biota.

## Introduction

The fire regime – typically characterized as the frequency, intensity, and seasonality of recurrent landscape fire (Gill 1981) – is an emergent property of the prevailing climate, biota, and ignitions (Bradstock 2010). As such, the fire regime is sensitive to direct influence by humans, who may alter ignition rates, fuel abundance and character, and fire behavior (e.g., active suppression) (Bowman et al. 2011). If fire regimes are substantially changed, ecosystems can experience abrupt shifts. For example, the introduction of exotic plant or animal species has been shown to dramatically alter fire frequency by changing fuel loads. Exotic shrubs and grasses can both increase fire frequency in closed forest (Fensham et al. 1994), and these shifts can lead to positive feedbacks that act to permanently reinforce the new ecosystem state (Russell-Smith and Bowman 1992). Hence, alternate stable state theory – that posits that strongly contrasting ecosystem states can exist permanently under identical exogenous environmental conditions – is now being widely applied to fire and vegetation ecology, a notable recent example being the fire-driven interchangeability of tropical savanna and closed forest biomes (Hirota et al. 2011; Staver et al. 2011; Murphy and Bowman 2012; Bowman et al. 2013).

That fire regimes can also exist as alternate stable states are illuminated by grass–fire cycles (D'Antonio and Vitousek 1992), where increases in fuel abundance, typically due to invasion by exotic grasses, lead to increases in fire frequency and intensity. This increases tree mortality and reduces tree cover, facilitating further increases in grass abundance, due to relaxation of competition for light. Hence, the invasive grass–fire cycle is an exemplar of how feedback processes can result in irreversible changes to fire regimes with knock-on effects to ecosystem properties and processes, such as reducing biodiversity (Brooks et al. 2004) and carbon storage (Bradley et al. 2006). Studying the effects of invasive grasses on fire regimes provides insights into the tolerance of ecosystems to particular fire intensities and frequencies. Grass–fire cycles can occur in natural systems where increased fire activity favors greater grass biomass, thereby increasing fire hazard. Indeed, the idea of “derived” savannas hinges on increased anthropogenic burning that allows the proliferation of grasses at the expense of woody cover. Examples of derived savannas have been reported around the tropics including New Guinea (Haberle 2007), Asia (Stott 1984), Africa (Greig-Smith 1991) and South America (Veldman and Putz 2011).

The application of alternate stable state theory to fire regimes and vegetation is relevant to understanding the changes that have taken place in northern Australia's tropical savanna landscapes in the last two centuries. In this region, there are significant and ongoing losses of biodiversity putatively related to changes in fire regimes associated with the transition from Aboriginal to European land management (Russell-Smith et al. 1998; Woinarski et al. 2011). There is anecdotal evidence that a natural grass–fire cycle has become established in parts of northern Australia, with increasing abundance of native *Sorghum* species (Miles 2003; Bowman et al. 2004, 2007a) and increased fire intensity and low spatial heterogeneity (Werner 2010). The recent expansion of high-biomass exotic grasses, such as gamba (*Andropogon gayanus*) has heightened concerns of a substantial intensification of the grass–fire cycle in northern Australian savannas (e.g., Rossiter et al. 2003).

**Figure 1:**
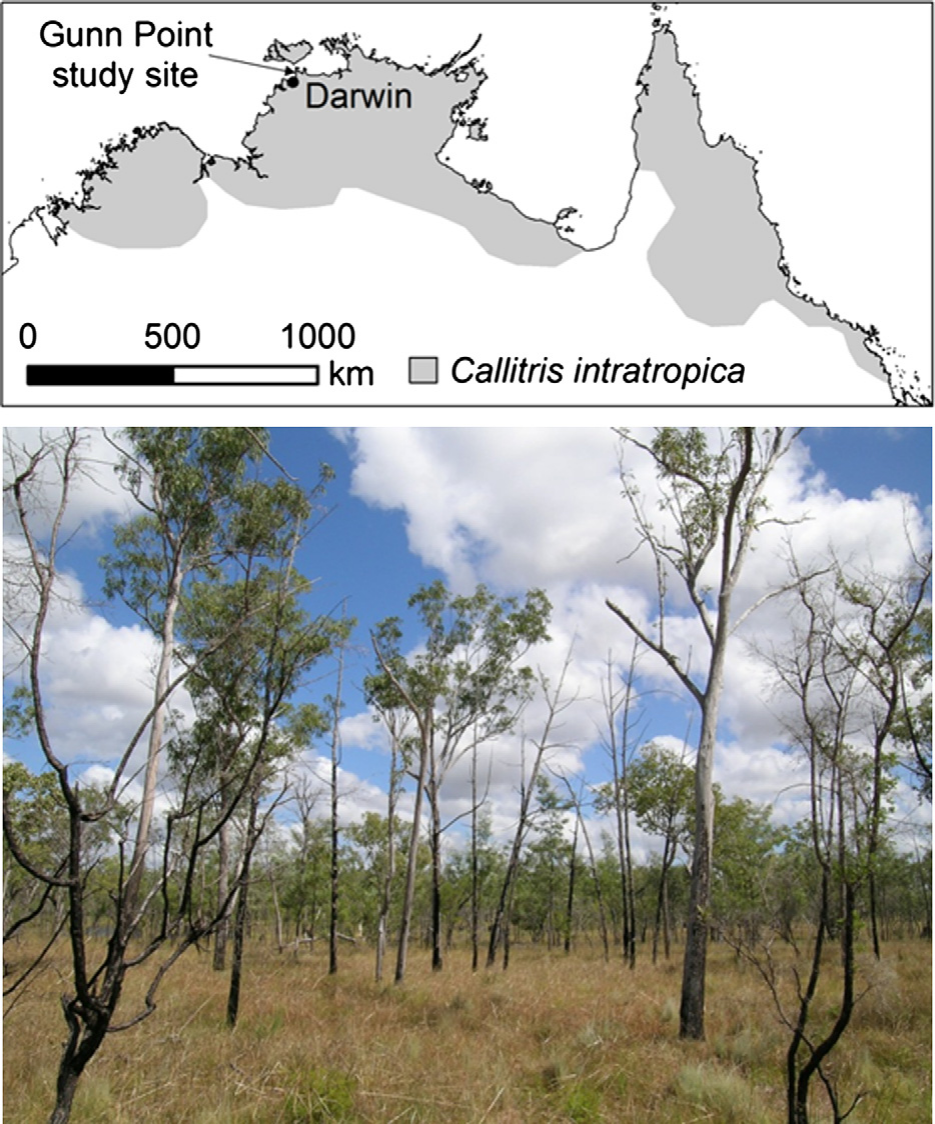
The location of the Gunn Point study area within the northern Australian range of *Callitris intratropica* (top panel), and a photograph of a fire-killed stand of *C. intratropica* (bottom panel; the remaining live trees are eucalypts).

A notable example of a natural grass–fire cycle is the collapse of the long-lived obligate-seeding conifer *Callitris intratropica* in many parts of northern Australia (Bowman and Panton 1993; Prior et al. 2011). Under Aboriginal fire management, the species occurs in small groves, where high canopy cover suppresses grass biomass, preventing the ingress of savanna fires which are fueled by a spare groundcover of short perennial grasses (Trauernicht et al. 2012). The groves provide fire-free sites to enable continuous recruitment of *C. intratropica*, as well as a range of other fire-sensitive woody plants (Trauernicht et al. 2013); however, increasing abundance of annual grasses has led to the breakdown of this feedback process in many areas, followed by rapid decline of *C. intratropica* populations (Bowman and Panton 1993) (Fig. 1).

Although *C. intratropica* is widely used as an example of the biological consequences of altered fire regimes, the mechanistic basis of this process has never been directly elucidated. We are able to test the grass–fire cycle hypothesis using as a model system an abandoned plantation of *C. intratropica* established in the 1960s on a cleared *Eucalyptus tetrodonta* savanna on deep lateritic soils at Gunn Point, northern Australia (Bowman 1986) (Fig. 1). *C. intratropica* occurs naturally in this area (Bowman and Wightman 1985). The aggressively invasive African grass, gamba (*Andropogon gayanus*), which was introduced to the region as fodder for cattle, has become established on the eastern edge of the plantation following the cutting of a power-line easement in the 2000s. This grass species is known to dramatically increase fine fuel loads and fire intensity (Setterfield et al. 2010). We document the impact of the fires in gamba grass invading the plantation on *C. intratropica* mortality and canopy architecture. We then report on experiments within the plantation to determine the effects of fires in different grass fuel loads, and experimental heating of trunks on tree canopies and tree survival.

## Materials and Methods

### Transects

Ten parallel transects, separated by at least 30 m, were established at right angles to a north-south road and power-line easement that form the edge of the *C. intratropica* plantation. Transects were positioned between the rows of mature *C. intratropica* trees spaced about 4 m apart. The four corners of 4 × 4 m quadrats were defined by trees, that is two pairs of adjacent trees (separated by about 4 m) on neighboring rows (also separated by about 4 m). The length of each transect was variable, being terminated at the point where there was no longer any grass biomass beneath *C. intratropica* trees.

Within a 1 m radius of the center of each quadrat, two hemispherical canopy cover photographs were taken using a digital camera with a fish-eye lens mounted 1.3 m above the ground on a tripod. The hemispherical photographs were analyzed to determine canopy cover. Directly beneath the tripod, a grass fuel sample was collected from a 0.5 × 0.5 m quadrat and weighed using a spring balance. For every fifth sample, a subsample (20–100 g) was collected, weighed, and stored in a paper bag. The moisture content of this subsample was determined gravimetrically after oven drying for 24 h at 70°C.

The two trees that defined the eastern side of each quadrat were scored as being either dead or alive. If alive, the proportion of foliage living was estimated in horizontal sections across the entire canopy width, at 2-m-height intervals.

### Grass fire experiment

Within the plantation, 71 quadrats measuring 8 × 8 m were established. Each quadrat was centered on four mature *C. intratropica* trees, and the diameter at breast height (130 cm; DBH) of each tree was recorded. In the center of each quadrat, hemispherical canopy photographs were taken in the wet season (January, 2012) (i.e., peak canopy condition). The quadrats were spatially separated such that the canopy photographs did not overlap. In the following dry season (June, 2012), six grass fuel treatments were allocated to the 71 quadrats. The treatments involved spreading dried hay at rates of 2 (*n* = 11), 5 (*n* = 13), 10 (*n* = 13), 20 t·ha^−1^ (*n* = 12), and a control treatment consisting of no hay (*n* = 22). The hay was then ignited from the perimeter of each quadrat using a drip-torch. No attempt was made to ignite the quadrats where no hay was spread. Meteorological data at the time of the fires were recorded using an automated weather station (Table S1). It is important to acknowledge that the structure of the hay, even when spread out by hand, potentially differs from that of grass that has remained in situ, most likely being of higher bulk density, and therefore less combustible (producing higher temperatures) but burning for a longer duration.

For each experimental fire, temperatures were characterized using an infrared thermometer aimed at a metal plate 1 m above the ground in the center of the quadrat, with temperature electronically logged every 1 sec. In the following dry season (July, 2013), survival of the trees was assessed, and repeat hemispherical photographs were taken to assess changes in canopy cover following the fires. For the four trees in each quadrat, the proportion of foliage living was estimated in 2 m horizontal sections of the canopy.

### Stem heating experiment

Forty six adult *C. intratropica* trees (mean DBH: 21 cm; mean bark thickness: 15 mm) in the plantation were selected for an investigation of their tolerance of exposure to high temperatures on the stem. A relatively low-temperature treatment consisted of wrapping a kerosene-soaked wick (2 cm wide) 14 times around the stem at ground level, for each of nine trees. The wick was ignited and continued burning until most kerosene was consumed; this took around 10 min. Thermocouples recorded maximum temperatures of >400°C on the outer surface of the bark. The remaining 37 trees were divided into three groups of 12–13 and a high-temperature treatment applied using butane burners (two per stem). These were used to heat the stems to 900°C for 30, 60, and 180 sec. Heat was applied to the whole circumference of the stem. Stem mortality was assessed 1 year later, and the proportion of foliage living was estimated in 2-m horizontal sections of the canopy.

To determine the insulating properties of the bark, 15 stems were cut down with a chainsaw and thermocouples inserted above the cambium and secured on the surface of the bark. Three gas burner treatments (30 sec, 2 min, 3 min) were applied, and the maximum temperatures on the bark surface and beneath the bark were recorded.

## Results

### Transects

**Figure 2:**
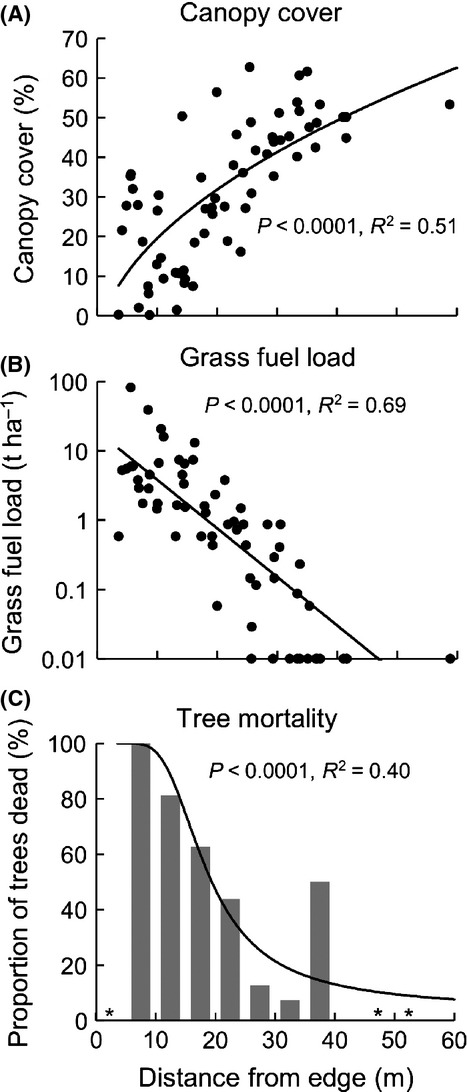
Variation in (A) canopy cover, (B) grass fuel load, and (C) tree mortality, with increasing distance from the surrounding open savanna toward into the interior of the *Callitris intratropica* plantation. The regression lines in (A) and (B) refer to least-squares linear regression models: cover∼sqrt(distance); and log(fuel + 0.01)∼distance. The regression line in (C) refers to a generalized linear model (binomial errors): proportion dead∼distance^−1^.The asterisks indicate an absence of data, rather than values of zero.

**Figure 3:**
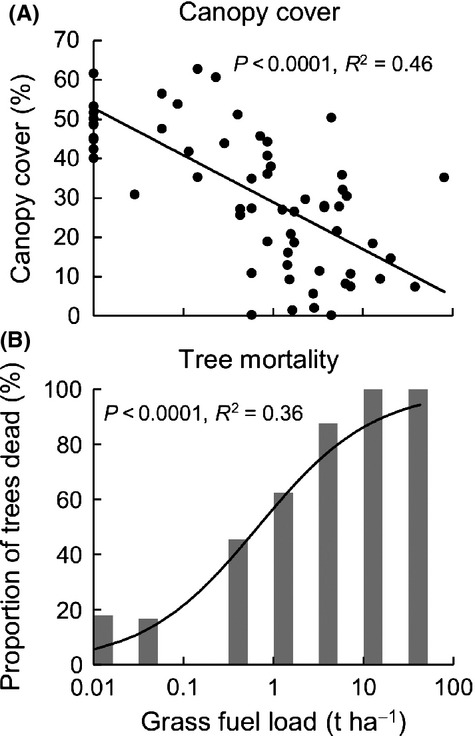
Relationships grass fuel load, and each of (A) canopy cover and (B) tree mortality along transects within the *Callitris intratropica* plantation. The regression line in (A) refers to a least-squares linear regression model: cover∼log(fuel + 0.01), and the regression line in (B) refers to a generalized linear model (binomial errors): proportion dead∼log(fuel + 0.01).

With increasing distance from the plantation-savanna boundary (i.e., moving from the savanna into the plantation), there was a steady increase in canopy cover (Fig. 2A). This accompanied a decrease in grass fuel load and frequency of dead trees (Fig. 2B–C). Hence, there was a clear negative relationship between grass fuel load and canopy cover, and a positive relationship between grass fuel load and the frequency of dead trees (Fig. 3). In areas with fuel loads exceeding 1 t·ha^−1^, the majority of trees were dead (Fig. 3B).

**Figure 4:**
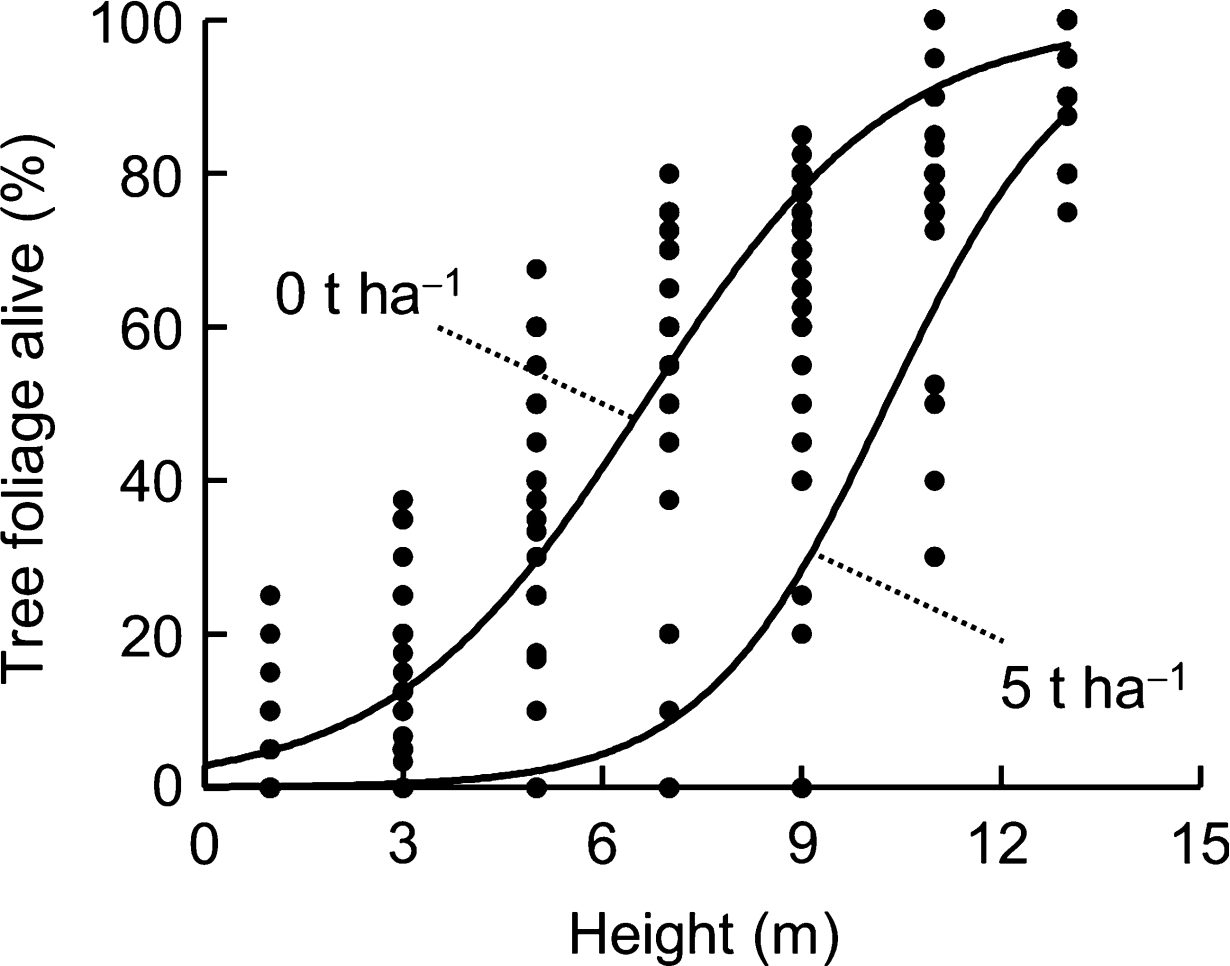
The relationship between live foliage distribution on surviving trees and grass fuel load, along transects within the *Callitris intratropica* plantation. The regression lines refer to a linear mixed effects model: logit(proportion alive)∼height * log(fuel + 0.01), with “transect position” as a random intercept. The effects of height, grass fuel load, and their interaction, were all highly significant (*P* < 0.0001, *P* < 0.0001, and *P* < 0.01, respectively; *R*^2^ = 0.69).

The vertical distribution of live foliage was strongly correlated with grass fuel load. At grass fuel loads of 5 t·ha^−1^, canopy foliage was “pruned” to small areas in the upper canopy (Fig. 4). With low fuel load, the canopy was dense in the lower half of the tree crown, with occasional branches reaching down to near the ground surface.

### Grass fire experiment

**Figure 5:**
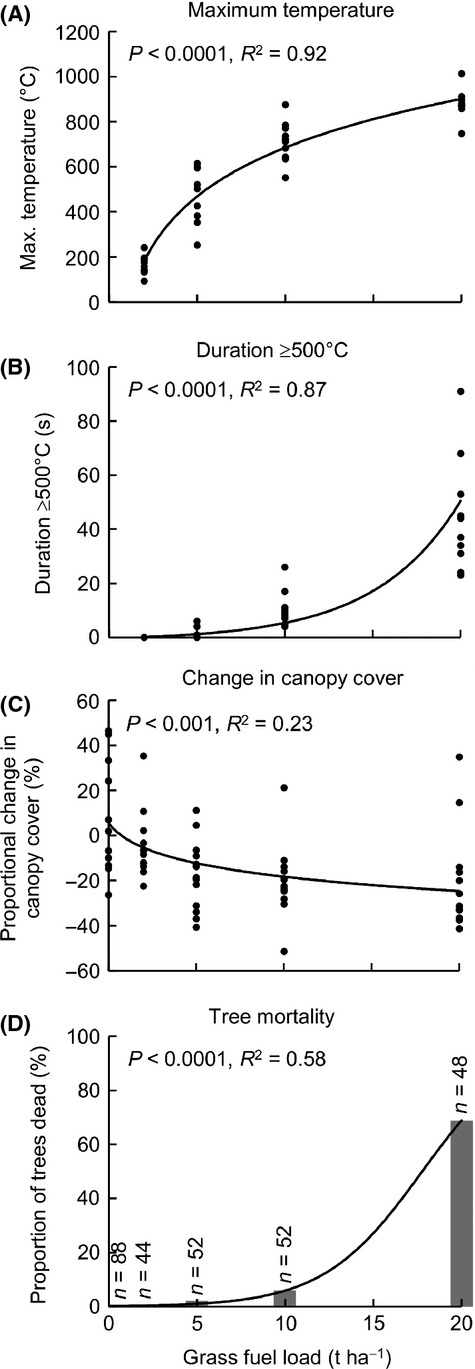
Results of the fire experiment involving artificially elevated grass fuel loads within the *Callitris intratropica* plantation. The regression lines in (A–C) refer to least-squares linear regression models: (A) maximum temperature∼log(fuel); (B) log(duration + 1)∼fuel; (C) proportional change in canopy cover∼log(fuel + 0.01). The regression line in (D) refers to a generalized linear model (binomial errors): proportion dead∼fuel.

The experimental grass fires were lit under very similar weather conditions (Table S1), yet the four fuel treatments produced fires of strongly contrasting intensities. Maximum temperature and duration of temperatures ≥500°C increased strongly with increasing fuel load (Fig. 5A-B).

**Figure 6:**
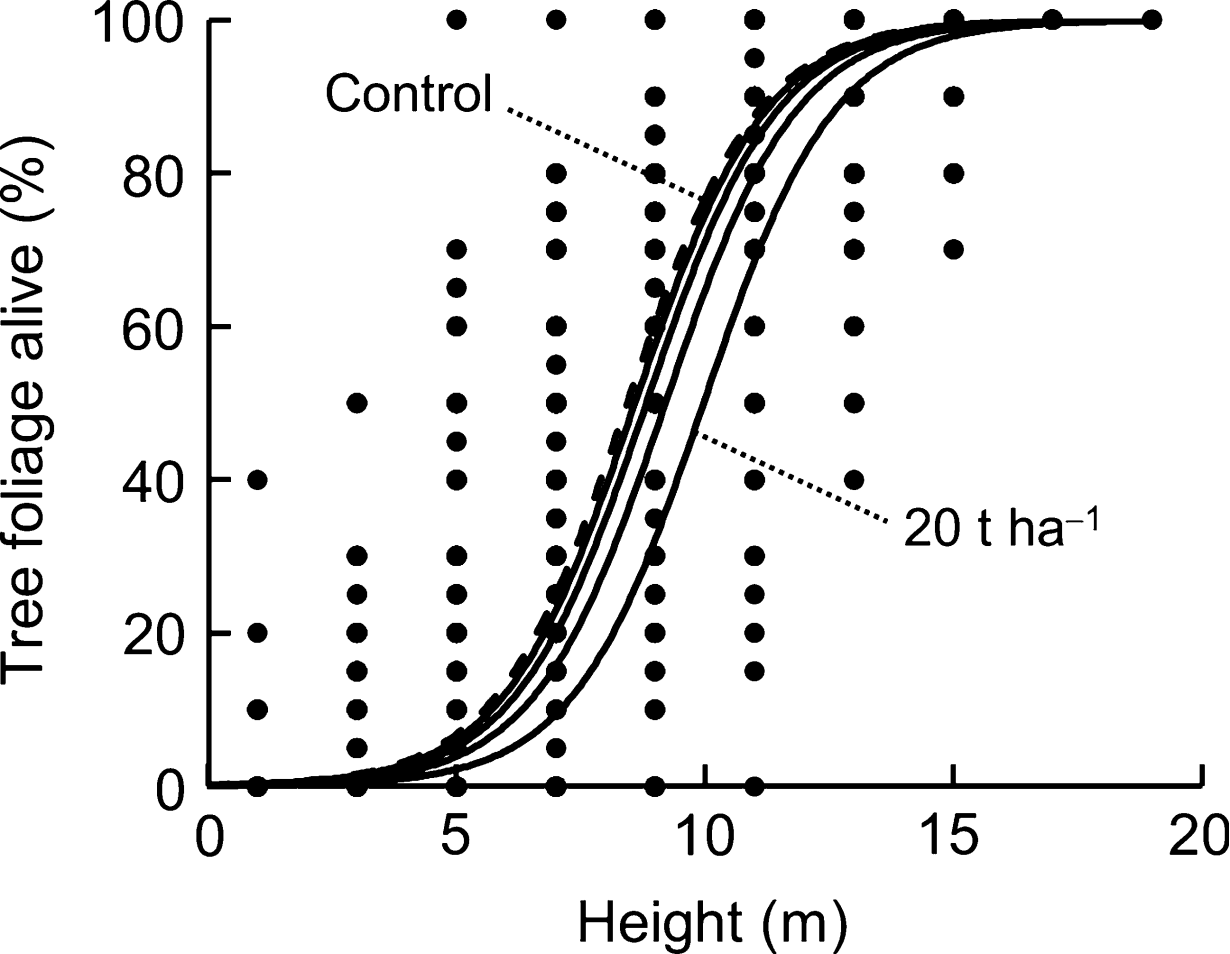
The relationship between live foliage distribution on surviving trees and grass fuel load used in the fire experiment. The regression lines refer to a least-squares linear regression model: logit(proportion alive)∼height * (fuel + 0.01). The effects of height and fuel load were highly significant (*P* < 0.0001 and *P* < 0.0001, respectively; *R*^2^ = 0.78).

Canopy cover was significantly affected by the experimental fires. The 2, 5, 10, 20 t·ha^−1^ treatments resulted in reductions in canopy cover of 5%, 12%, 18%, and 25%, respectively (Fig. 5C). These changes in canopy cover due to experimental fires are much smaller in magnitude than the differences observed along the savanna-plantation transects, where an increase in grass fuel load from 0 to 20 t·ha^−1^ was accompanied by a proportional reduction in canopy cover of 75% (Fig. 3A), implying that either canopy cover is lost through successive fires, and/or the experimental fires were of lesser intensity than typical wildfires, possibly due to the relatively high bulk density of the spread hay. The impact of the experimental fires on the foliage was concentrated in the crown below 12 m (Fig. 6), and these patterns broadly matched those observed on the transects although magnitude of the change was less after a single fire.

Mortality of adult trees following the experimental fires increased rapidly with increasing fuel load (Fig. 5D). There was a very marked jump from 6% mortality at a fuel load of 10 t·ha^−1^ to 69% mortality at 20 t·ha^−1^.

### Stem heating experiment

There was no mortality of adult *C. intratropica* trees exposed to >400°C temperatures for approximately 10 min (Table 1). There was minimal (17%) mortality for the 30 sec gas burner treatment, with outside-bark temperatures reaching 865°C ± 54 (95% CI) and inside-bark temperatures reaching just 55°C ± 10. Mortality increased substantially for the 2 and 3 min heat treatments, although the maximum temperatures were not substantially greater – 909°C ± 95 and 897°C ± 42 outside-bark, and 89°C ± 6 and 90°C ± 7 inside-bark, respectively. Mortality was 85% and 100% for the 2 and 3 min gas burner treatments, respectively. The stem heating treatments had no discernible effect on canopy foliage of surviving trees.

**Table 1:** The impact of four experimental heat treatments, applied to the trunks of mature *Callitris intratropica* individuals, on whole-tree mortality. Mortality was highly significantly related to type of treatment (Pearson's chi-squared test, *P* < 0.0001). Maximum temperature refers to temperatures measured outside the bark in the area where heat was applied.

Treatment	Duration (min:sec)	Maximum temperature (°C ± 95% CI)	*n*	Tree mortality (%)
Kerosene-soaked wick	10:42	406 ± 51	9	0
Gas burner	0:30	865 ± 54	12	17
Gas burner	2:00	909 ± 95	13	85
Gas burner	3:00	897 ± 42	12	100

## Discussion

We have used a plantation of the northern Australian conifer *Callitris intratropica* invaded by an African grass to help disclose the processes that are causing a region-wide population collapse of this iconic obligate-seeding tree. Our results show that a grass–fire cycle associated with the invasion of a high-biomass African grass (gamba, *Andropogon gayanus*) resulted in the reduction in *C. intratropica* plantation canopy cover due to both tree death and restriction of foliage to the top-most branches. High grass fuel loads (>5 t·ha^−1^) are associated with significant tree death. Tree death appears due to the cumulative effect of canopy loss, associated with successive fires which also increase grass fuel loads. We reach this conclusion because single experimental grass fires, using similar fuel loads (though higher bulk density, and that burn for a longer duration) as observed at the grass invasion front, resulted in significantly less canopy loss and tree death than observed at the invasion front.

The experimental heating of *C. intratropica* trees using kerosene wicks and gas burners suggests that the mechanism causing tree death is the loss of foliage rather than exposing the cambium to lethal temperatures. Trees could only be killed by exposing trunks to temperatures over 900°C for about 100 times longer than the duration achieved in grass fires (ca. 1 sec). Briefer exposures over 900°C or longer exposures (>10 min) at more moderate temperatures (>400°C) did not kill mature *C*. *intratropica* individuals. The tolerance of *C. intratropica* trunks to high temperatures, also noted by Lawes et al. (2011), underscores the insulating capacity of the species’ thick bark. We found that exposure of the surface of trunks to 900°C for 30 sec only resulted in under-bark heating to 55°C ± 10 (95% CI), which is below the lethal temperature for cambium (around 60°C: van Mantgem and Schwartz 2003), although exposure for 2 and 3 min caused cambial temperatures to become lethal (89°C ± 6 and 90°C ± 7, respectively), consistent with other studies of conifers (e.g., Gutsell and Johnson 1996; Dickinson and Johnson 2004). The nonlethal stem heating treatments had no effect on canopy architecture, a result that is consistent with the interpretation that grass fires kill *C. intratropica* trees by damaging the canopy (typically through cumulative impacts) rather than by cambial death. This explanation is consistent with Ryan et al. (2010), who point out that a key cause of mortality following fire is the destruction of buds in the canopy.

Our results are consistent with the hypothesis that a switch from low-intensity surface fires in litter and sparse perennial grasses to high biomass of annual grasses following the transition from Aboriginal to European fire regimes has resulted in widespread population collapse of *C. intratropica* (Bowman et al. 2004, 2007a) (Table 2). Such a switch increases the mortality of *C. intratropica* because of the cumulative impact on the species’ canopies, rather than via topkill. This interpretation is supported by studies on an Aboriginal clan estate in central Arnhem Land with large healthy populations of *C. intratropica*, where the fire regime remains characterized by low-intensity surface fires in grass fuel loads much lower than similar areas under European fire management (Yibarbuk et al. 2001; Bowman et al. 2007a). In this area, Aboriginal fire management creates spatially variable burning patterns and a fine-grained mosaic of fire histories (Bowman et al. 2004). Such patchy burning enables the establishment of small groves of *C. intratropica* that have dense litter mats which are far less flammable than adjacent savannas (Bowman and Wilson 1988). The *C. intratropica* groves provide safe sites for regeneration of a suite of fire-sensitive taxa (Trauernicht et al. 2012, 2013). A feature of *C. intratropica* trees in clumps is the spreading foliage that often reaches to the ground surface. In contrast, single adult *C. intratropica* trees occur in frequently burnt savannas, that have a distinctive canopy architecture of foliage being restricted to the upper branches (Trauernicht et al. 2012). Under European management, *C. intratropica* populations are collapsing due to the absence of seedling establishment and high adult mortality rates (Prior et al. 2007). That frequent fires create a “recruitment bottleneck” is well established for this species, which is particularly sensitive to fire in the juvenile stage (Bowman and Panton 1993; Russell-Smith 2006; Prior et al. 2007).

**Table 2:** Published observations to support the hypothesis that a grass–fire cycle is driving the decline of *Callitris intratropica* other fire-sensitive elements of the biota across northern Australia.

Observation	Citation
1. Biodiversity declines (granivorous birds, small mammals, obligate-seeder heaths) across northern Australia, putatively associated with changes to fire regimes	Russell-Smith et al. (1998), Franklin et al. (2005), Woinarski et al. (2011)
2. *C. intratropica* in widespread decline	Bowman and Panton (1993), Prior et al. (2007), Graham (2001)
3. Adult *C. intratropica* resistant to high fire frequencies, but easily killed by moderate-intensity fires	Bowman et al. (1988), Trauernicht et al. (2012)
4. Biomass of annual grasses elevated in areas under European fire management, compared to Aboriginal-managed lands	Yibarbuk et al. (2001), Bowman et al. (2004, 2007a)
5. High biomass of annual grasses increases fire intensity and decreases fine-scale patchiness of fires	Yibarbuk et al. (2001), Werner (2010)
6. Fire activity occurs earlier in the fire season in recent decades, signaling greater contribution of rapidly-curing grasses to fuel	Bowman et al. (2007b)

## Conclusion

In this study, we have used an extreme case of a high-biomass African grass invading a plantation of a native Australian fire-sensitive conifer to draw a parallel with a more subtle native grass–fire cycle that is suspected of driving the decline of the same tree species across much of its range. We argue that in addition to causing a recruitment bottleneck (Prior et al. 2007), a key mechanism of the grass–fire cycle in eliminating *C. intratropica* adults is the cumulative degradation of the canopy, reducing the photosynthetic capacity of individual trees, eventually leading to tree death. The cause of the canopy loss appears to be related to the very limited capacity of scorched *C. intratropica* crowns to recover, a feature typical of conifers (Harrington 1993; Stephens and Finney 2002; Ryan et al. 2010; Clarke et al. 2013), in marked contrast to eucalypts (Burrows 2002), which dominate northern Australian savannas. Furthermore, because *C. intratropica* has a low CO_2_ assimilation rate per unit of leaf mass (*A*_mass_) compared to other (broadleaved) savanna trees (Prior et al. 2003), a significant reduction in canopy volume due to fire may limit subsequent tree growth to a greater extent than in other savanna trees.

Although our findings are directly applicable to just a single tree species, *C. intratropica*, they do highlight that the management challenge of reversing the fire-driven decline of savanna biodiversity in northern Australia hinges on reducing grass biomass to switch fire regimes from high-intensity grass fires, with rapid rates of spread and flame heights that reach to the midstorey, to low-intensity litter fires with low rates of spread and lower flame heights. We suggest that this is unlikely to be achieved with the current management objective of reducing typical fire intensities through the extensive use of prescribed burning under mild fire weather conditions (e.g., Russell-Smith et al. 2013). Increases in grass biomass and resultant fire intensities may explain ongoing biodiversity declines in Kakadu National Park despite substantial changes in the predominant season of fire (from predominantly late dry season, under severe fire weather conditions, to predominantly early dry season, under mild fire weather conditions). Such switching of fire regimes can potentially be achieved by burning immediately after the first wet season rains, thereby killing the cohort of immature annual grasses (e.g., Lonsdale et al. 1998), or use of grazing herbivores to reduce the abundance of grass (Bowman 2012). Indeed, Trauernicht et al. (2013) noted an association between feral buffalo abundance and the health of *C. intratropica* populations in central Arnhem Land. Our study also highlights the risk to tree cover in northern Australian savannas posed by fires in areas heavily infested with high-biomass African grasses. Such fires have the potential to drive rapid transformation of the savannas by eliminating fire-sensitive trees and causing the eventual death of more fire-resistant trees, including eucalypts, through recurrent defoliation (e.g., Ferdinands et al. 2006).

Our study highlights that humans have the significant capacity to modify fire regimes by altering fuel arrays, resulting in substantial knock-on effects on biodiversity. In our case, the driver is grass biomass, resulting in a positive feedback with fire intensity thereby amplifying the difference between Aboriginal and European fire management. Elsewhere in the world, there are examples of land use causing changed fire regimes that both positively and negatively affect tree species abundance. For example, in north American *Pinus ponderosa* forests, there has been a change from savanna woodlands with low-intensity surface fires burning in grass, to dense forests with episodic high-intensity crown fires following the shift from Native American to European management (Mast et al. 1999). The reintroduction of burning in *Pinus palustris* savannas in the southeastern United States, where there is a long history of fire suppression, has been shown to cause high mortality apparently because of killing cambium at the base of trees by smoldering fires in deep accumulations of organic matter (“duff layers”) (Varner et al. 2005). In contrast, overgrazing on tall grass prairies on the Great Plains has resulted in the establishment of dense *Juniperus* woodlands that exclude fire due to the absence of sufficient fine fuels (Twidwell et al. 2013). There is also circumstantial evidence that prehistoric fire regimes switched following the loss of Pleistocene megafauna in both North America (Gill et al. 2009) and Australia (Rule et al. 2012): in these cases, it is asserted that a proliferation of woody biomass fueled more intense fires. Understanding the capacity of humans to modify fuel arrays is key to devising sustainable fire management, necessitating a shift in thinking from simply manipulating the season and frequency of savanna burning (e.g., Russell-Smith et al. 2013) to understanding how best to manipulate fuels (Bowman 2012). A concrete example is the assisted expansion of rainforests in anthropogenically derived savannas by mechanical suppression of grass (King et al. 1997).

## Supporting Information

**Table S1.** Mean values (±95% CI) for a range of meteorological variables affecting fire behavior, measured during the fire experiment.
