# Supplementary material for: A grass–fire cycle eliminates an obligate-seeding tree in a tropical savanna
Source: Ecol Evol. 2014 Oct 14;4(21):4185–94. doi: 10.1002/ece3.1285 (PMC4242569; doi:10.1002/ece3.1285)
Supplement: Supplementary file 1 — Table S1. Mean values (±95% CI) for a range of meteorological variables affecting fire behavior, measured during the fire experiment. [file ece30004-4185-SD1.docx]

Table S1. Mean values (±95% CI) for a range of meteorological variables affecting fire behaviour, measured during the fire experiment. Analysis of variance suggested no significant differences between the treatment means (*P*>0.05), for any of the variables measured.

| Fuel load  (t ha^−1^) | Mean wind speed  (km h^−1^) | | | Maximum gust speed (km h^−1^) | | | Temperature  (°C) | | | Relative humidity  (%) | | | *n* |
| --- | --- | --- | --- | --- | --- | --- | --- | --- | --- | --- | --- | --- | --- |
| 2 | 0.2 | ± | 0.1 | 1.9 | ± | 0.4 | 27.5 | ± | 2.0 | 50.8 | ± | 8.5 | 11 |
| 5 | 0.3 | ± | 0.1 | 2.3 | ± | 0.5 | 26.8 | ± | 5.2 | 42.9 | ± | 9.2 | 9 |
| 10 | 0.2 | ± | 0.1 | 2.1 | ± | 0.3 | 27.9 | ± | 1.5 | 50.4 | ± | 4.9 | 11 |
| 20 | 0.2 | ± | 0.1 | 2.0 | ± | 0.6 | 26.7 | ± | 2.2 | 47.4 | ± | 8.8 | 10 |
